# miR-34a Inhibits Proliferation and Invasion of Bladder Cancer Cells by Targeting Orphan Nuclear Receptor HNF4G

**DOI:** 10.1155/2015/879254

**Published:** 2015-03-23

**Authors:** Huaibin Sun, Jun Tian, Wanhua Xian, Tingting Xie, Xiangdong Yang

**Affiliations:** ^1^Department of Hemodialysis, Qilu Hospital, Shandong University, Jinan 250012, China; ^2^Department of Nephrology, Qilu Hospital, Shandong University, Jinan 250012, China

## Abstract

miR-34a is a member of the miR-34 family and acts as a tumor suppressor in bladder cancer. This study explored the regulative role of miR-34a on an orphan nuclear receptor HNF4G, which has a well-confirmed role in bladder tumor growth and invasion. qRT-PCR analysis was applied to measure miR-34a expression in two tumorigenic bladder cancer cell lines 5637 and T24 and one normal human urothelial cell line SV-HUC-1. Luciferase assay was performed to verify the putative binding between miR-34a and HNF4G. The influence of miR-34a-HNF4G axis on cell viability, colony formation, and invasion was assessed with loss- and gain-of-function analysis. This study observed that the miR-34a expressions in 5637 and T24 cells were significantly lower than in SV-HUC-1, while the muscle invasive cell sublines 5637-M and T24-M had even lower miR-34a expression than in the nonmuscle invasive sublines. HNF4G has a 3′-UTR binding site with miR-34a and is a direct downstream target of miR-34a. miR-34a can directly downregulate the expression of HNF4G and thus inhibit tumor cell viability, colony formation, and invasion. Therefore, miR-34a-HNF4G axis is an important pathway modulating cell viability, proliferation, and invasion of bladder cancer cells.

## 1. Introduction

Bladder cancer is a common urinary malignant tumor characterized as high recurrence rate. Approximately 70% of the patients had at least one recurrence within 5 years [1]. At diagnosis, about 30% bladder cancer cases were muscle invasive [2]. The high-grade muscle-invasive cases are associated with higher risk of metastasis, even after radical cystectomy and chemotherapy [1, 3]. Therefore, it is necessary to explore the underlying molecular mechanisms of bladder cancer growth and invasion.

MicroRNAs (miRNAs) are a group a conservative, small (19–25 nucleotides in length), and noncoding RNAs repressing translation or promoting degradation of target mRNA through binding to the complementary sequences in the 3′ untranslated region (3′-UTR) [4]. miRNAs can be either tumor suppressors or oncogenes depending on their target genes in different cancers [5, 6]. Previous studies reported that miR-34a is a number of the miR-34 family and acts as a tumor suppressor in multiple cancers, including bladder cancer [7–9]. In fact, miR-34a is the most significantly regulated downstream miRNA of the p53 pathway and its expression influences cell apoptosis, cell-cycle arrest, senescence, and cancer cell chemosensitivity [10–13]. However, the downstream targets of miR-34a in bladder cancer are still not well recognized. Recently, some studies reported that nuclear receptors (NRs) such as Nur77 (NR4A1) and Nurr1 (NR4A2) were involved in bladder cancer progression [14, 15]. One recent study observed that orphan NR HNF4G was remarkably upregulated in bladder cancer and this upregulation gives rise to bladder cancer progression through promoting cancer cell growth and invasion [16].

In this study, we explored the regulative network of miR-34a in bladder cancer and firstly reported that HNF4G is a direct downstream target of miR-34a. miR-34a could suppress tumor cell proliferation and invasion through suppressing HNF4G expression.

## 2. Methods

### 2.1. Cell Culture

The human bladder cancer cell lines 5637 and T24 and normal human urothelial cell line SV-HUC-1 were maintained in RPMI-1640 medium (GIBCO) containing 10% fetal bovine serum, 100 U/mL penicillin, and 100 mg/mL streptomycin. All cells were incubated in humidified air with 5% CO_2_ at 37°C. Cells in logarithmic phase of growth were harvested for all experiments in this study.

### 2.2. Isolation of Muscle Invasive and Nonmuscle Invasive Tumor Cell Sublines

Six-well Transwell cell culture inserts with 8-*μ*m pores (Corning) were used to isolate muscle invasive and nonmuscle invasive 5637 and T24 cell sublines. Generally, 5 × 10^5^ cells were firstly cultured in serum-free RPMI-1640 medium for 24 h and then suspended and seeded into the top chamber coated with 200 mg/mL of Matrigel (BD Biosciences). To stimulate cell penetration with a chemotactic gradient, the lower well was filled with 2.5 mL RPMI-1640 medium containing 20% bovine serum. 24 h after incubation, the noninvasive cells on the topside and the invasive cells on the bottom side of the polycarbonate membrane were harvested, respectively, for further cultivation. After 10 rounds of selection, the stable invasive cell sublines were designated as 5637-M and T24-M and the stable noninvasive sublines were designated as 5637-NM and T24-NM, respectively.

### 2.3. Quantitative Real-Time PCR (qRT-PCR)

Total RNA from cell samples were extracted with TRIzol isolation reagent (Invitrogen) according to recommended instructions. The concentration and purity of the RNA samples were measured with a UV-visible spectrophotometer (NanoDrop Technologies). Taqman miRNA Assays were used to quantify mature miR-34a expression. RNU6B was used as an internal control. Quantification was performed in triplicate.

### 2.4. Vector Preparation

The human HNF4G lentiviral expression vector (RC219703L2) and the empty control vector were purchased from OriGene. To produce sufficient lentiviral particles for transfection, HEK 293T cells were cotransfected with expression vector or control vector and the Lenti-vpak packaging kit (Origene) by using Lipofectamine 2000 (Invitrogen). 48 h after transfection, the viral supernatant with titer around 10^6^–10^7^ TU/mL was further used for transfection.

Full-length HNF4G cDNA purchased from OriGene was subcloned into the eukaryotic expression vector pcDNA3.1 (+) to generate HNF4G wide type (wt) expression vector. HNF4G mutant (mut) expression vector (without miR-34a specific 3′-UTR binding site) was generated with the QuikChange Multi Site-Directed Mutagenesis kit (Stratagene) according to the manufacturer's protocol.

### 2.5. miRNA Mimic, Inhibitors and siRNA

Chemically synthesized miR-34a mimics, the oligonucleotides miR-34a inhibitor, HNF4G siRNA, and the corresponding negative controls were all purchased from Life Technologies.

### 2.6. Cell Transfection

5637-M cells were transfected with HNF4G siRNA (50 nM) for HNF4G knockdown, or transfected with miR-34a mimics (50 nM) for miR-34a overexpression, or cotransfected with human HNF4G pcDNA3.1 expression vector (wt or mut) and miR-34a mimics by using Lipofectamine 2000 (Invitrogen). 5637-NM cells were transfected with miR-34a inhibitor (100 nM) for miR-34a knockdown by using Lipofectamine 2000 (Invitrogen) or treated with HNF4G lentiviral supernatant under the presence of 5 *μ*g/mL Polybrene (Sigma-Aldrich) for HNF4G overexpression.

### 2.7. Colony Formation Assay

5 × 10^2^ cells after transfection were seeded in one well of six-well cell culture plate and further incubated for 10 days without disturbance. After that, cell colony formations were visualized by staining with 0.1% crystal violet. The rate of colony formation was calculated by using the following equation: colony formation rate = (number of colonies/number of seeded cells) × 100%. Each test was performed in triplicate.

### 2.8. Cell Viability Assay

24 h after transfection, 5 × 10^3^ cells were plated to one well of 96-well plates and further cultured. At 24, 48, or 72 h, culture medium was removed and cell-counting solution (WST-8, Dojindo Laboratories) was added to the wells. After one-hour incubation at 37°C, the absorbance at 450 nm of the solution was detected with MRX II (Dynex Technologies, Chantilly, VA, USA). Each test was performed with five repeats.

### 2.9. *In Vitro* Invasion Assay

24-well Transwell cell culture inserts with 8 *μ*m pores (Corning) was used to assess the invasive ability of the cell sublines. 5 × 10^4^ cells firstly cultured in serum-free RPMI-1640 medium for 24 h was suspended and seeded into the top chamber coated with 200 mg/mL of Matrigel (BD Biosciences). To stimulate cell penetration with a chemotactic gradient, the lower well was filled with 2.5 mL RPMI-1640 medium containing 20% bovine serum. After 24 h incubation at 37°C, the topside of the membrane was wiped with cotton wool to remove noninvasive cells. The invading cells on the bottom side were fixed by using 100% methanol for 10 min and then dried under room temperate and stained with 0.1% crystal violet for 20 min. The number of cells was counted under a microscope with 100x magnification. Each test was performed in triplicate.

### 2.10. Western Blot Analysis

48 h after transfection, the cells were lysed in cell lysis buffer. The total protein concentration of the lysates was determined with the BCA Protein Assay kit (Pierce) according the manual. Protein sample (30 *μ*g) was separated on 10% SDS-PAGE and then transferred onto nitrocellulose membrane. Membranes were blocked with 5% nonfat dried milk for 1 h and then incubated overnight with primary antibody. After three times washing with 1x TBS buffer containing 0.1% Tween 20, the membrane was further incubated with corresponding secondary antibody for 1 h. Signal intensities were detected using the Odyssey Infrared Imaging System (Li-Cor Biosciences). The primary antibodies and the working concentrations were mouse anti-HNF4G (R&D system, Clone N3224, 1 *μ*g/mL) and mouse anti-GAPDH (R&D system, AF5718, 1 *μ*g/mL). The second antibody used was Horseradish peroxidase-conjugated anti-mouse IgG (R&D system, BAF007, 1 : 5000).

### 2.11. Dual-Luciferase Assay

To generate luciferase reporter vectors, a pair of oligonucleotide with wide type 3′-UTR region of HNF4G (containing binding site with miR-34a) were designed and synthesized (F: 5′-cATTATATTTTTATATACTGAACTGCCATAGATGCTGGAAAAAAGGAAg-3′; R: 5′-tcgacTTCCTTTTTT  CCAGCATCTA  ACCGTCATCA  GTATATAAAA  ATATAATgagct-3′) (RiboBio, Guangzhou, China). The oligonucleotide pair was annealed by heating at 90°C for 3 min and then at 37°C for 15 min. Then the duplex was inserted between the* SacI* and* SalI* sites of the pmirGLO Dual-Luciferase miRNA Target Expression Vector (Promega). This expression constructed was designated as Luc-HNF4G. The same method was applied to generate Luc-HNF4G-mut with mutant 3′-UTR region of HNF4G by using the following oligonucleotide pair: F: 5′-cATTATATTTTTATATACTGATGACGGTTAGATGCTGGAAAAAAGGAAg-3′; R: 5′-tcgacTTCCTTTTTTCCAGCATCTAACCGTCACAGTATATAAAAATATAATgagct-3′. The insertion was verified by sequencing. miR-34a mimics or the control was cotransfected with Luc-HNF4G or Luc-HNF4G-mut into HEK 293T cells. Cells were lysed 48 h after transfection. Luciferase activity of the lysates was detected by using the Dual-Light luminescent reporter gene assay (Applied Biosystems).

### 2.12. Statistical Analysis

Experimental data are presented as mean ± SE based on the results of at least three repeats. Between group comparisons was all based on Student's *t*-test. *P* < 0.05 was considered as significant difference.

## 3. Results

### 3.1. miR-34a Expression Is Decreased in Bladder Cancer Cells and Further Decreased in Muscle Invasive Sublines

**Figure 1:**
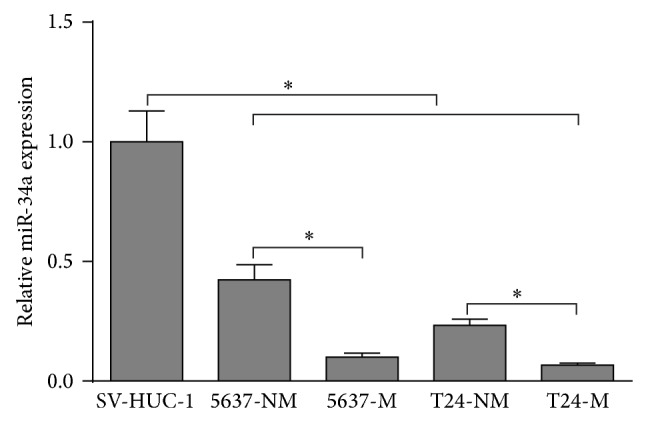
miR-34a expression is decreased in bladder cancer cells and further decreased in muscle invasive sublines. qRT-PCR analysis of miR-34a expression in muscle invasive and nonmuscle invasive sublines of tumorigenic 5637 and T24 bladder cancer cells and in normal human urothelial cell line SV-HUC-1. Each bar represents the relative fold change compared to SV-HUC-1 cell line. Fold change was calculated by 2^−ΔΔCt^. ^*^ *P* < 0.05, ^**^ *P* < 0.01. Error bars depict s.e.m.

In order to identify miR-34a expression pattern in bladder cancer cells, we explored its expression in muscle invasive and nonmuscle invasive sublines of two tumorigenic bladder cancer cells (5637 and T24) and one normal human urothelial cell line SV-HUC-1. miR-34a expression was significantly lower in both sublines of 5637 and T24 cells than in SV-HUC-1 cells (Figure 1). More importantly, even within the same cell line, the muscle invasive cell sublines 5637-M and T24-M had significantly lower miR-34a expression than the corresponding nonmuscle invasive sublines (5637-NM and T24-NM, resp.) (Figure 1). These results suggested that miR-34a expression is decreased in bladder cancer cells and further decreased in muscle invasive sublines.

### 3.2. miR-34a Inhibits Cell Viability, Colony Formation Rate, and Invasion of Bladder Cancer Cells

**Figure 2:**
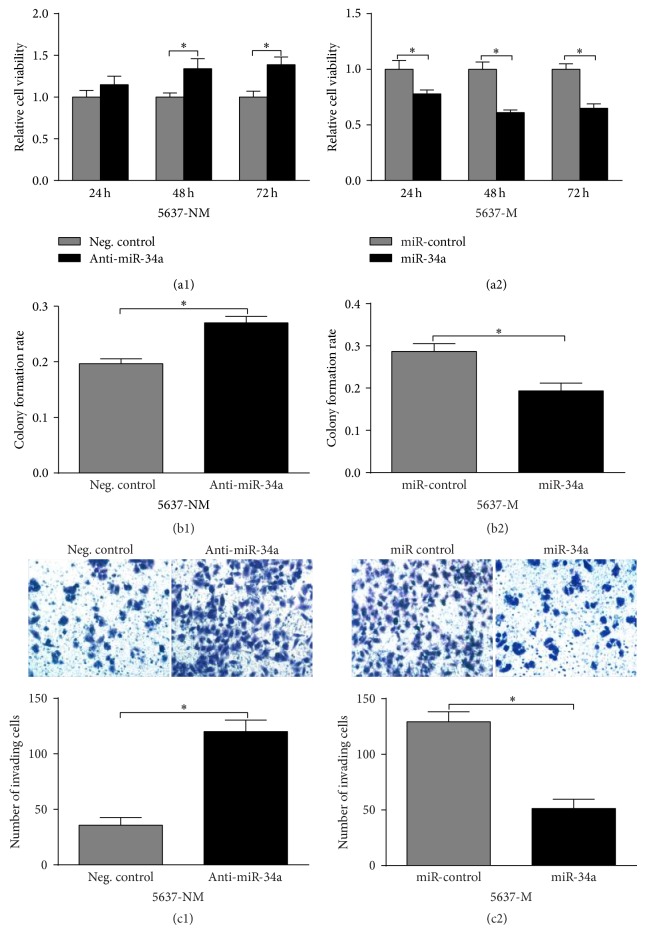
miR-34a inhibits cell viability, colony formation rate, and invasion of bladder cancer cells (a) MTT assay of cell viability. Knockdown (a1) and overexpression (a2) of miR-34a caused significantly promotion and inhibition of cell viability, respectively, in a time dependent manner. (b) Colony formation assay. Knockdown of miR-34a caused 37.3% increase of colony formation rate in 5637-NM cells (b1), while overexpressing miR-34a reduced it by 32.6% in muscle invasive counterpart (b2). (c) Transwell assay of cell invasion. The cell invasion assay indicated that miR-34a knockdown in 5637-NM cells increased cell invasion (c1) while overexpressing miR-34a in 5637-M cells inhibited cell invasion (c2). ^*^ *P* < 0.05. Error bars depict s.e.m.

5637-M cells and 5637-NM cells were transfected with miR-34a mimics and inhibitors, respectively. In 5637-NM cells, miR-34a knockdown led to significantly increased cell viability at 48 and 72 h (Figure 2(a1)). In 5637-M cells, miR-34a overexpression was associated with significantly decreased cell viability at 24, 48, and 72 h (Figure 2(a2)). Similar trends were also observed in colony formation rate and invasion. miR-34a inhibition resulted in significantly higher colony formation rate (Figure 2(b1)) and invasion (Figure 2(c1)) in 5637-NM cells, while its overexpression led to significantly lower colony formation rate (Figure 2(b2)) and invasion (Figure 2(c2)) of 5637-M cells. These results suggest that miR-34a can inhibit cell viability, colony formation rate, and invasion of bladder cancer cells.

### 3.3. HNF4G Is a Downstream Target of miR-34a in Bladder Cancer

**Figure 3:**
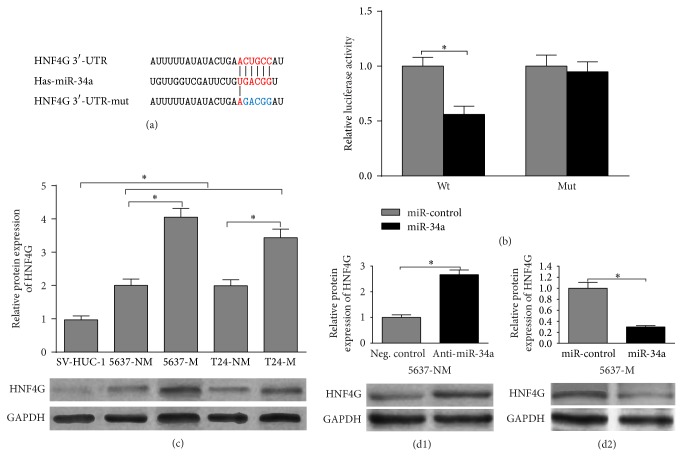
HNF4G is a downstream target of miR-34a in bladder cancer. (a) A predicted seed region (red) in the 3′-UTR of HNF4G was shown (top). The mutated sequence used was highlighted in blue (bottom). (b) HEK293T cells were cotransfected with either 50 nM miR-34a mimics or NC oligos and 200 ng plasmid carrying either Wt or Mut 3′-UTR of HNF4G. The relative firefly luciferase activity normalized with Renilla luciferase was measured 48 h after transfection. (c) Western blot analysis showed that the HNF4G protein levels in bladder cancer cells were inversely correlated with miR-34a expression as in Figure 1. (d) Knockdown of endogenous miR-34a remarkably increased HNF4G expression at protein level, while ectopic overexpression significantly decreased HNF4G expression in bladder cancer cells. ^*^ *P* < 0.05. Error bars depict s.e.m.

Through comparison in TargetScan (http://www.targetscan.org/), we identified a putative binding site between miR-34a and 3′-UTR of HNF4G and then designed a mutant sequence (Figure 3(a)). Transfection of miR-34a mimics significantly inhibited the relative luciferase activity of luc-HNF4G-wt, but not luc-HNF4G-mut in HEK293T cells (Figure 3(b)). In addition, HNF4G presented an inverse expression trend to miR-34a. HNF4G expression was significantly higher in 5637 and T24 cells than in SV-HUC-1 cells (Figure 3(c)). In addition, within the tumor cell lines, its expression was also higher in muscle invasive sublines than in nonmuscle invasive subline (Figure 3(c)). To further confirm the influence of miR-34a on HNF4G expression, HNF4G expression in 5637-M cells with miR-34a overexpression and in 5637-NM cells with miR-34a inhibition was measured. Western blot analysis revealed that miR-34a knockdown led to increased HNF4G expression (Figure 3(d1)), while miR-34a overexpression was associated with decreased HNF4G expression (Figure 3(d2)). There results suggest that miR-34a can directly target HNF4G and modulate its expression.

### 3.4. mirR-34a Inhibits HNF4G Expression to Reduce Bladder Cancer Proliferation and Invasion

**Figure 4:**
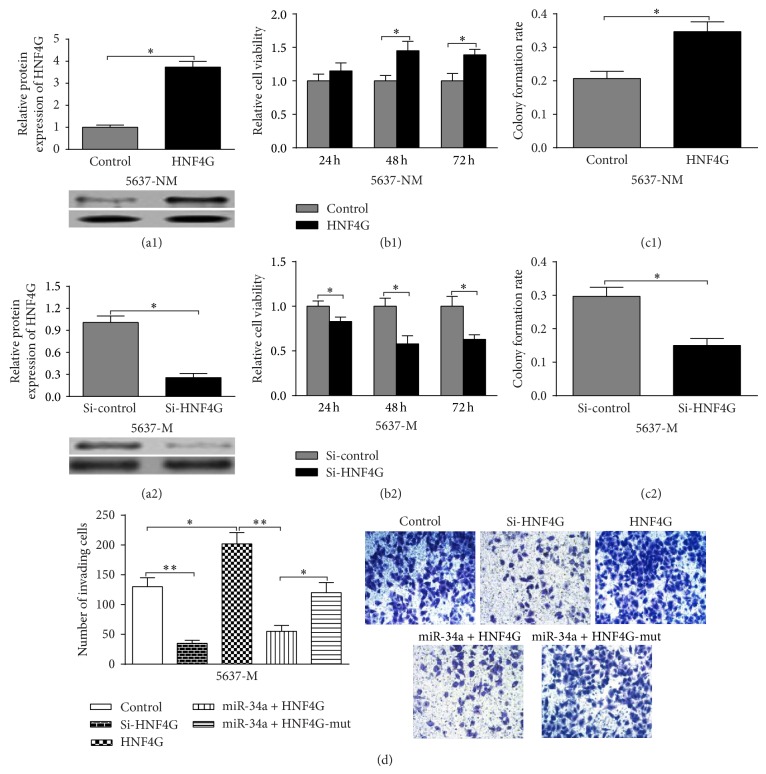
miR-34a inhibits HNF4G expression to reduce bladder cancer proliferation and invasion. (a) Western blot analysis of HNF4G expression in 5637-NM (a1) and 5636-M (a2) cells with HNF4G overexpression and knockdown, respectively. (b) MTT assay showed that overexpressing HNF4G in 5637-NM (b1) and knockdown in 5637-M (b2) cells promoted and suppressed cell growth, respectively. (c) HNF4G promoted colony formation. Overexpressing HNF4G caused 67.8% increase of colony formation rate in 5637-NM cells (c1), while inhibiting its expression reduced the rate by 49.4% in invasive counterpart (c2). (d) The cell invasion assay indicated that knockdown of HNF4G in 5637-M cells inhibited cell invasion while overexpressing it enhanced cell invasion. miR-34a significantly abrogated HNF4G-wt induced higher cell invasion but had no effect on HNF4G-mut induced cell invasion increase. ^*^ *P* < 0.05, ^**^ *P* < 0.01. Error bars depict s.e.m.

To explore the role of HNF4G in bladder cancer, 5637-NM and 5637-M cells were transfected for HNF4G overexpression and knockdown, respectively (Figures 4(a1) and 4(a2)). 5637-NM cells with HNF4G overexpression had significantly higher viability at 48 and 72 h (Figure 4(b1)), while the 5637-M cells with HNF4G knockdown had significantly lower viability at 24, 48, and 72 h (Figure 4(b2)). Similar trend was observed in colony formation rate. HNF4G overexpression was associated with significantly increased colony formation rate in 5637-NM cells (Figure 4(c1)), while HNF4G inhibition led to significantly decreased rate in 5637-M cells (Figure 4(c2)). As to cell invasion, miR-34a significantly abrogated HNF4G-wt induced higher cell invasion but had no effect on HNF4G-mut induced cell invasion increase (Figure 4(d)). These results suggested that the inhibitive role of miR-34a in bladder cancer cells is directly related to its regulation over HNF4G expression.

## 4. Discussion

There is accumulative evidence showed that lower miR-34a expression is involved in several types of cancers, including colon, ovarian, and prostate cancer [17–19]. In bladder cancer, miR-34a is generally considered as a tumor suppressor [8, 9]. Inactivation of miR-34a by CpG methylation leads to increased risk of bladder cancer [7]. miR-34a expression is also lower in high histological grade and high clinical stage tumors than in the lower grade or stage cases [9]. These findings suggested that miR-34a might be related to tumor development and progression. In the current study, we also observed lower miR-34a expression in tumorigenic bladder cancer cells (5637 and T24) than in the normal human urothelial cell line (SV-HUC-1) and even lower expression in the muscle invasive bladder tumor cell sublines (5637-M and T24-M) than in the nonmuscle invasive sublines (5637-NM and T24-NM). These findings confirmed that lower miR-34a expression is a risk factor of bladder cancer. However, how aberrant miR-34a expression affect bladder cancer progression is still not fully understood.

Previous studies already confirmed that miR-34a is a downstream target of p53 and can induce apoptosis and G1-arrest [11, 20]. Due to its important role in p53 pathway, its expression level is highly related to chemosensitivity of bladder cancer cells [12, 21]. But only limited studies reported the downstream targets of miR-34a in bladder cancer. One recent study reported that miR-34a could inhibit cell migration and invasion of invasive urothelial bladder carcinoma through targeting Notch1 [8]. Other studies reported that miR-34a could affect chemotherapy sensitiveness of tumor cancer cells by targeting multiple downstream genes in p53 signaling pathway, including Sirt-1, CDK6, E2F3, and Bcl-2 [10, 11, 20]. However, due to the complex regulative network of miRNAs, it is highly possible that miR-34a can modulate multiple cellular pathways simultaneously in bladder cancer.

The current study observed that miR-34a could reduce bladder tumor cell viability, colony formation rate, and invasion. In addition, we also verified HNF4G as a new direct target of miR-34a. NRs are a set of transcription factors regulating gene expression spatiotemporally and thus mediating cell differentiation, homeostasis, and metabolism [22]. However, their endogenous ligands and biological functions are not clearly identified [23]. In bladder cancer, one recent study found that HNF4G is a most frequently upregulated gene and its overexpression could promote bladder cancer growth and invasion through regulating the hyaluronan synthase 2 gene [16]. Our study confirmed the oncogenic role of HNF4G in bladder tumor. HNF4G overexpression could significantly increase tumor cell viability, colony formation rate, and invasion, while HNF4G knockdown could achieve reverse effects in* in vitro* model. More importantly, we also identified one of its upstream regulators and described a novel mechanism of miR-34a in regulating bladder cancer development. miR-34a can directly target 3′-UTR of HNF4G and regulate its expression. Functional study showed that miR-34a-HNF4G axis could modulate tumor cell viability, colony formation rate, and invasion. In fact, the regulative network of miRNAs is quite complex, we could not exclude the possibility that other miRNAs are involved in the regulation of HNF4G in bladder cancer [24] or miR-34a could modulate bladder cancer through other pathways. However, considering the significant effect of miR-34a-HNF4G axis in modulating cell viability, proliferation, and invasion of bladder cancer cells, this axis at least is one of the most important regulative paths.

## 5. Conclusion

In conclusion, miR-34a is a tumor suppressor in bladder cancer. miR-34a-HNF4G axis is an important pathway modulating cell viability, proliferation, and invasion of bladder cancer cells. However, due to the complex regulative network of miR-34a, further study is required to explore other mechanisms of miR-34a in bladder cancer.
